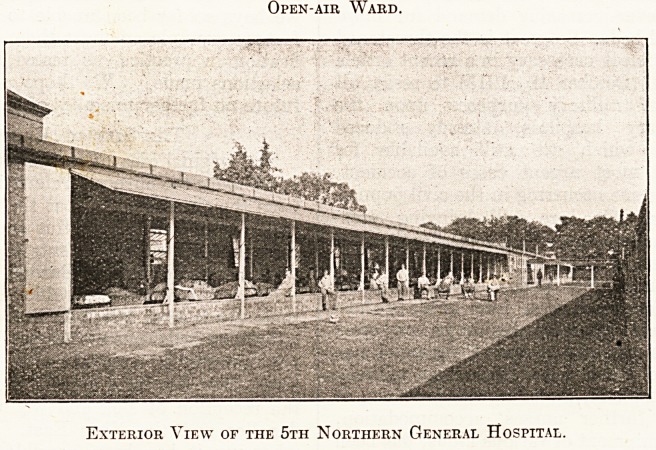# Poor-Law Buildings and Military Hospitals

**Published:** 1915-10-23

**Authors:** 


### POOR-LAW BUILDINGS & MILITARY HOSPITALS.

From the first we have advocated the occupation of Poor-Law establishments, which were suitable for the purpose, as temporary military hospitals. We have steadily fought the battle of the Poor-Law infirmary and its efficiency as a hospital, structurally and administratively. We hold that Poor-Law infirmaries of the best type should be renamed State hospitals, and in them should be found a good proportion of the additional hospital beds which the passing of the National Insurance Acts has rendered necessary to the adequate treatment in all circumstances of the insured. The intervention of the great war has swept aside for the moment considerations of this character, and the civil population has to face much suffering, and even danger to permanent health and possibly life itself, by the diminution of facilities for treating members of the civil population in up-to-date hospitals, as and when occasion arises.

### Some Difficulties and a Problem.

The circumstances of the war as it progresses have caused an ever-increasing demand for more and more hospital beds. So great is the threatened pressure that we felt it necessary in a recent article in The Hospital (October 16, 1915) to resist all further raids for military purposes upon the beds in voluntary hospitals, already reduced to a minimum, which are still available for the use of the most urgent cases of accident, operation, and disease occurring in the civil population. The War Office are still bringing pressure to bear upon the Poor-Law authorities to make arrangements for the transfer of existing sick accommodation under the control of the Guardians for conversion into military hospitals. Poor-Law infirmary buildings are often excellent, and the expenditure upon them which has been necessary to make them efficient as military and Territorial hospitals, including the provision of operation units, further nurses' accommodation, and up-to-date equipment of various kinds, has been relatively small. In the case of some Poor-Law infirmaries the difficulties presented have been increased by the necessity to provide adequate boundaries so as to enforce discipline, and render impossible the soldiers' habit of treating ordinary fences, after experience of trench warfare, as no check in fact to their wanderings outside and away from the area which represents the site of the Poor-Law infirmary. This circumstance has added not a little to the difficulties of enforcing discipline and to the work of those responsible for the administration and order of this type of temporary military hospital. Another problem has arisen from the limitations of the site and the not infrequent absence of any provision for a recreation hall or for sports and games and other means of exercise, requiring ample space, whereby the military patients can be kept amused and in growing health. Of course there are Poor-Law infirmaries, like that recently built at North Evington, near Leicester, where there is abundant space, and where full facilities exist of the kind just referred to. Where a fine site like that at North Evington has been obtained by the Poor-Law authorities, one objection to a Poor-Law building for military purposes—namely, the necessity for extension and enlargement—is overcome.

### Unsuitable Buildings.

We have thought it to be essential to the proper understanding of the position created by the ever-growing demand for temporary military hospital accommodation throughout the country to point out the limitations of very many Poor-Law establishments, good as most of the accommodation thus provided undoubtedly is. We have already shown why it is undesirable, if it is not even wrong, as it certainly would be extravagant, to commandeer elementary school buildings, technical and art schools, and other large buildings like town halls, institutes, theatres, and music-halls, which at first it was considered might, in case of emergency, be converted for use as a portion of the Territorial hospital system. In a few cases some of this type of buildings have been occupied as hospitals, and we have so far been unable to find a single satisfactory example amongst them of success or justification in practice, as tested by results, for the selections made. We hope and believe that in future no further mistakes of the kind will occur.

### The Size of Hospitals.

The original idea of the selection of buildings as possible for the purposes referred to may have arisen from the circumstance that there are relatively few really large hospitals in this country, though there are enormous establishments of the kind to be met with in various parts of Europe. We have seen in a Russian hospital a ward which contained 500 beds; there are several hospitals in Germany which contain 2,000 beds, one even more; and within the last few months a hospital with accommodation for 3,000 beds has been rapidly provided at Malta for the reception of sick and wounded from the Dardanelles and the Near East. In the same way, wherever it has been possible, the tendency has been for the War Office authorities to sanction, and even to encourage, considerable additions to the bed accommodation of existing military, Territorial, and other hospitals, so that the number of beds now available in several cases is infinitely greater than those to be met with in any civil hospital in Great Britain. All these points are important when the provision of an adequate supply of hospital beds in these islands for sick and wounded sailors and soldiers is under consideration.

### Mental Institutions as Territorial Hospitals.

This brings us to consider next the most important and, on the whole, probably the most successful type of buildings which the War Office has been able to commandeer for the purposes of military hospital accommodation. We allude to the county and other large asylums or mental hospitals, several of which have been converted into military hospital purposes with the completest success. Each of the mental hospitals we refer to is placed upon a magnificent site of most ample proportions, which includes extensive and beautiful gardens, recreation grounds and abundant vacant space on which can be erected temporary hospital buildings for the accommodation of almost any number of patients, say, up to 5,000. Most people are genuinely and increasingly interested in war conditions and the efforts which are being made to meet all demands in very varying directions. It would be an education, however, to many people, and a most useful experience, if they were to make it their business to obtain a permit and to visit one of the new temporary military hospitals which were formerly county asylums or mental hospitals. There is no better way than this to understand and appreciate the vastness of the requirements, where the completest arrangements exist to fulfil all the conditions involved in perfecting the discipline, providing for the special needs of the moment in regard to our sick and wounded warriors, and placing them under conditions which do, in fact, leave nothing to desire in the direction of means to secure the rapid restoration to health and the fullest recovery of the sick and wounded patients. We have been speaking so far of existing county asylums and mental hospitals which have been taken over by the military authorities, but we must now conclude this portion of our subject by enforcing once more the wisdom of leaving to the local as opposed to the central authority in London, the finding of suitable accommodation or old buildings which may be unoccupied, and their adaptation for hospital purposes.

Open-air Ward.

Exterior View of the 5th Northern General Hospital.

### The Importance of Business Methods.

The 5th Northern General Hospital is a case in point. At the beginning of the war the nucleus of this hospital was old asylum buildings, the property of the County Council, with extensive and attractive grounds and grass-land which had been left unoccupied for years. We gave an account of the transformation of these buildings into a military unit of 520 beds in The Hospital of October 10, 1914. Since then this base hospital has been extended by the erection of temporary buildings, of which we give extensive illustrations, making provision for 530 additional beds. This extension was agreed to by Sir Alfred Keogh and the War Office in preference to using a number of elementary school and other buildings under the circumstances we have explained earlier. This is the second time that proposals to convert the technical and art schools and two other schools for the purposes of providing hospital beds for the wounded under plans which had actually been approved by the War Office have been rejected, in consequence of the urgent representations made by the authorities at Leicester of the serious interference with the educational organisation in the town which otherwise must have resulted. We commend this fact to the principal authorities and to the inhabitants of other great centres of population within the United Kingdom. It is a striking proof of the power, economical value, and vast importance and influence of business methods in war-time. A visit to the 5th Northern General Hospital would well repay representatives from every town and county where difficulties have arisen in regard to the extension of existing hospital accommodation for the wounded.

### Well Done, Board of Control!

The Leicester plan has effected much saving in capital expenditure, and the adaptation of the old asylum and the further extensions have secured a maximum of economy. Further, and of equal importance, have been the administrative and health results of the utilisation of the grounds and adjacent land connected with the old asylum. Few Territorial or military hospitals can compare with these results, and in every case the Board of Control's action in co-operating with the War Office to enable asylum buildings and grounds to be utilised for hospital purposes in connection with the war is a fact which confers credit on all concerned, and has secured results for which the nation can never be sufficiently grateful. The great hospitals which have been built up in the neighbourhood of Birmingham, at Sheffield, and elsewhere by the utilisation of asylum buildings are as remarkable as they are satisfactory. Let us leave the schools to the children, the art and technical schools to the students, and places of amusement and municipal buildings to fulfil their proper uses. Experience has shown the extravagance and impropriety of attempting to utilise as hospitals buildings which cannot be spared and ought not to be diverted from their original purpose. It has brought us better and more economical methods of meeting in the best manner every demand for additional hospital beds for the wounded and the sick.

## Figures and Tables

**Figure f1:**